# Omega-3 fatty acids decrease oxidative stress and inflammation in macrophages from patients with small abdominal aortic aneurysm

**DOI:** 10.1038/s41598-019-49362-z

**Published:** 2019-09-10

**Authors:** Lara T. Meital, Mark T. Windsor, Maria Perissiou, Karl Schulze, Rebecca Magee, Anna Kuballa, Jonathan Golledge, Tom G. Bailey, Christopher D. Askew, Fraser D. Russell

**Affiliations:** 1 Centre for Genetics, Ecology & Physiology, School of Health and Sport Sciences, University of the Sunshine Coast, Sippy Downs, Qld Australia; 2 VasoActive Group, School of Health and Sport Sciences, University of the Sunshine Coast, Sippy Downs, Qld Australia; 3 Sunshine Vascular, Buderim, Qld Australia; 4 Sunshine Coast University Hospital, Birtinya, Qld Australia; 5 Queensland Research Centre for Peripheral Vascular Disease, College of Medicine and Dentistry, James Cook University, Townsville, Australia; 6 Department of Vascular and Endovascular Surgery, Townsville Hospital, Townsville, Australia; 7 Centre for Research on Exercise, Physical Activity and Health, School of Human Movement and Nutrition Sciences, The University of Queensland, St. Lucia, Qld Australia

**Keywords:** Fatty acids, Monocytes and macrophages

## Abstract

Abdominal aortic aneurysm (AAA) is associated with inflammation and oxidative stress, the latter of which contributes to activation of macrophages, a prominent cell type in AAA. Omega-3 polyunsaturated fatty acids (n-3 PUFAs) have been reported to limit oxidative stress in animal models of AAA. The aim of this study was to evaluate the effect of the n-3 PUFA docosahexaenoic acid (DHA) on antioxidant defence in macrophages from patients with AAA. Cells were obtained from men with small AAA (diameter 3.0–4.5 cm, 75 ± 6 yr, n = 19) and age- matched male controls (72 ± 5 yr, n = 41) and incubated with DHA for 1 h before exposure to 0.1 µg/mL lipopolysaccharide (LPS) for 24 h. DHA supplementation decreased the concentration of tumour necrosis factor-α (TNF-α; control, 42.1 ± 13.6 to 5.1 ± 2.1 pg/ml, p < 0.01; AAA, 25.2 ± 9.8 to 1.9 ± 0.9 pg/ml, p < 0.01) and interleukin-6 (IL-6; control, 44.9 ± 7.7 to 5.9 ± 2.0 pg/ml, p < 0.001; AAA, 24.3 ± 5.2 to 0.5 ± 0.3 pg/ml, p < 0.001) in macrophage supernatants. DHA increased glutathione peroxidase activity (control, 3.2 ± 0.3 to 4.1 ± 0.2 nmol/min/ml/μg protein, p = 0.004; AAA, 2.3 ± 0.5 to 3.4 ± 0.5 nmol/min/ml/μg protein, p = 0.008) and heme oxygenase-1 mRNA expression (control, 1.5-fold increase, p < 0.001). The improvements in macrophage oxidative stress status serve as a stimulus for further investigation of DHA in patients with AAA.

## Introduction

The long-chain omega-3 polyunsaturated fatty acids (n-3 PUFAs), docosahexaenoic acid (22:6n-3; DHA) and eicosapentaenoic acid (20:5n-3, EPA), have been suggested to have cardioprotective^1,2^, anti-inflammatory^3^, immunoregulatory^4^, antioxidant^5,6^ and anti-tumour activities^7^. These beneficial effects on human health have been attributed to (a) competition with arachidonic acid (AA) for the enzymes involved in the biosynthesis of pro-inflammatory mediator molecules^8^, (b) suppression of pro-inflammatory nuclear factor kappa B (NF-*κ* B) via modulation of toll-like receptor 4 (TLR4) signalling^9^ and activation of peroxisome proliferator-activated receptor gamma (PPAR γ)^10^, (c) activation of the G-protein-coupled receptor free fatty acid receptor 4 (FFA4, formerly GPR120)^11^ and d) metabolism to pro-resolution lipid mediators (e.g. resolvins, protectins, maresins)^12,13^. In synergism with their anti-inflammatory and pro-resolution activities, n-3 PUFAs have recently been documented to suppress pro-oxidant activity by upregulating genes encoding cytoprotective antioxidant proteins such as heme oxygenase 1 (HO-1) and glutathione peroxidase (GPx)^14–16^. HO-1 is a single, transmembrane 32-kDa protein that plays a central role in stress adaptation^17^. HO-1 provides cells and tissues with an inducible antioxidant defence mechanism that can be ubiquitously activated in response to elevated levels of heme, its natural substrate, and a multiplicity of endogenous factors such as heavy metals, cytokines, hormones, growth factors, nitric oxide and endotoxins^17^. The enzyme’s robust ability to defend against oxidative insults is linked to the removal of reactive, pro-oxidant heme and to the biological effects of biliverdin, bilirubin, and carbon monoxide, the three reaction products^18^. The GPx family includes eight phylogenetically related antioxidant enzymes (GPxs 1–8) with essential, location-specific physiological effects involving the reduction of H_2_O_2_ to water and the detoxification of organic hydroperoxides to their corresponding alcohols^19^. By readily decomposing peroxides generated under a variety of physiological conditions, GPx enzymes play a vital role in limiting the strength and extent of reactive oxygen signals and contribute importantly to oxidation-reduction homeostasis and cellular protection^20^.

While n-3 PUFAs have been suggested to limit adverse outcomes associated with inflammation, oxidative stress and disturbed antioxidant status, a deeper understanding of the therapeutic potential of these bioactive nutrients is required in patient populations with conditions characterised by inflammation and oxidative overload such as abdominal aortic aneurysm (AAA). This study aimed to evaluate the effect of DHA on antioxidant defence in macrophages from patients with and without AAA. The HO-1 inhibitor, SnPP (10 μM), and FFA4 receptor antagonist, AH7614 (2 μM), were used to examine mechanisms underlying n-3 PUFA effects.

## Results

### DHA inhibited 8-isoprostane production

**Figure 1:**
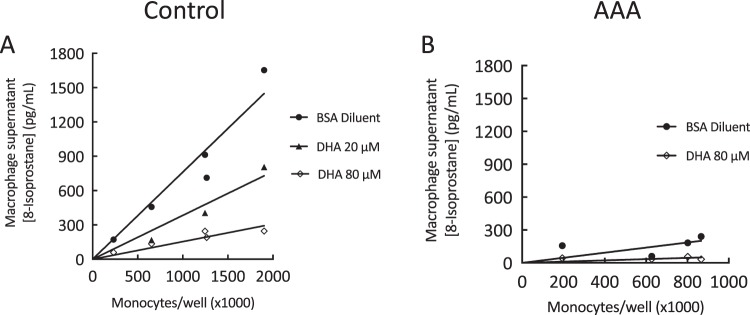
Concentration of 8-isoprostane in supernatants of monocyte-derived macrophages obtained from control participants and AAA patients. Monocytes were isolated from whole blood, grown in culture and, following spontaneous differentiation into macrophages, exposed to a 24 h incubation with 0.1 μg/ml lipopolysaccharide (LPS) prior to collection of the cells and supernatants. LPS-stimulated 8-isoprostane production (pg/ml), plotted against total number of circulating monocytes present in whole blood samples, was lower in AAA (**B**) compared to control macrophage supernatants (**A**). DHA decreased LPS-stimulated 8-isoprostane production in a dose-dependent manner in control participant macrophages. A non-significant trend for decreased 8-isoprostane production was observed in macrophages from AAA patients.

Incubation of cells with DHA decreased LPS-stimulated 8-isoprostane production in a dose-dependent manner in macrophages from healthy control participants (Fig. 1A). The slope for non-DHA treated data (BSA diluent; R^2^ = 0.93) was 7.3-fold greater than that of the DHA 80 μM data (R^2^ = 0.83, p = 0.002). Although LPS-stimulated 8-isoprostane production was suppressed in macrophages from the AAA patient cohort, a trend towards a further decrease in 8-isoprostane levels was observed when these cells were incubated with DHA (Fig. 1B).

### DHA uniformly inhibited cytokine production

**Figure 2:**
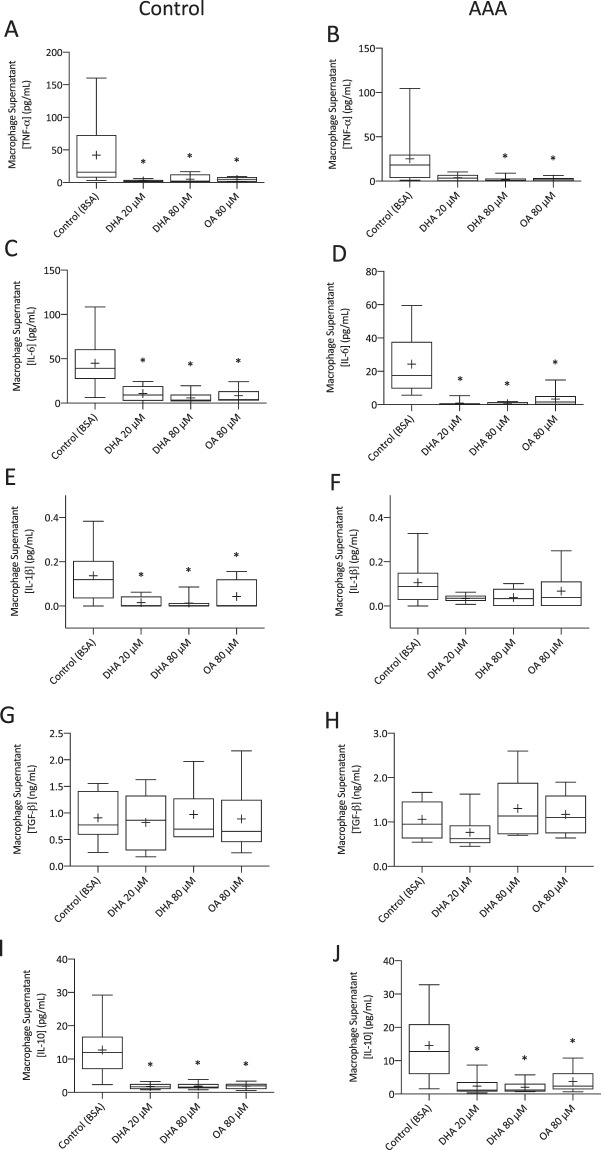
Cytokine concentrations in macrophage supernatants obtained from control participants and AAA patients. Control DHA-supplemented non-stimulated macrophages (20 μM n = 5–9; 80 μM, n = 7–10) produced lower levels of tumour necrosis factor alpha (TNF-α; **A**), interleukin-6 (IL-6; **C**), interleukin-1 β (IL-β; **E**) and interleukin-10 (IL-10; **I**) compared to non-supplemented macrophages. DHA supplementation of AAA non-stimulated patient macrophages (20 μM n = 6–7; 80 μM, n = 8–10) decreased the production of TNF-α (**B**), IL-6 (**D**) and IL-10 (**J**). A trend toward decreased levels of IL-β (**F**; 20 μM n = 7; 80 μM n = 10) was observed. Transforming growth factor-β (TGF-β) levels in both control (**G**) and AAA (**H**) macrophages were unaffected by DHA supplementation. *p < 0.05.

DHA-supplemented macrophages from control participants produced lower levels of TNF-α (Fig. 2A; 20 μM p < 0.001, 80 μM p = 0.004), IL-6 (Fig. 2C; 20 μM p = 0.002, 80 μM p < 0.001), IL-1 *β* (Fig. 2E; 20 μM p = 0.005, 80 μM p = 0.004) and IL-10 (Fig. 2I; 20 μM p = 0.004, 80 μM p = 0.002) compared to non-supplemented cells. In AAA patient macrophages, DHA supplementation suppressed the production of TNF-α (Fig. 2B; 80 μM p = 0.006), IL-6 (Fig. 2D; 20 μM p < 0.001, 80 μM p < 0.001) and IL-10 (Fig. 2J; 20 μM p = 0.008, 80 μM p = 0.004). A non-significant decrease was observed for IL-1 β (Fig. 2E). DHA supplementation did not alter TGF-β levels in macrophages from either the control (Fig. 2G) or AAA (Fig. 2H) cohorts. The effects of oleic acid on cytokine levels were similar to those of DHA. While CHD was evident in two thirds of the AAA cohort, no significant difference in cytokine concentrations (IL-6, IL-1β, TNF-α, IL-10, TGF-β) was observed for these patients when compared to non-CHD patients.

### DHA increased GPx activity in monocyte-derived macrophages

**Figure 3:**
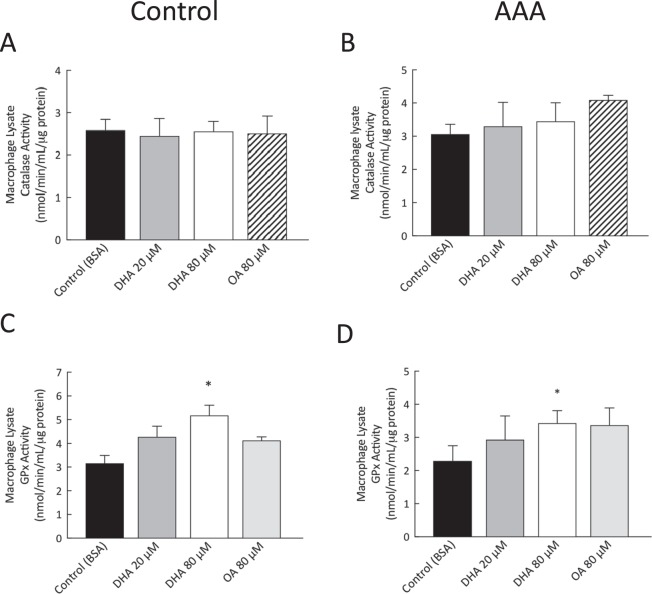
Antioxidant enzyme activity levels in control participant and AAA patient monocyte-derived macrophage lysates. Macrophage catalase activity was unaffected by DHA supplementation in control participants (**A**) and AAA patients (**B**). DHA supplementation increased glutathione peroxidase (GPx) activity in non-stimulated macrophages from control participants (**C**) and patients with AAA (**D**). Catalase and GPx activity and expression levels were unaffected by supplementation with OA. *p < 0.05.

DHA supplementation had no effect on catalase activity in macrophages obtained from control (Fig. 3A) or AAA participants (Fig. 3B). GPx activity was significantly increased in control and AAA macrophages supplemented with 80 μM DHA (control Fig. 3C, p = 0.005; AAA, Fig. 3D, p = 0.008).

### DHA increased HO-1 expression in monocyte-derived macrophages

**Figure 4:**
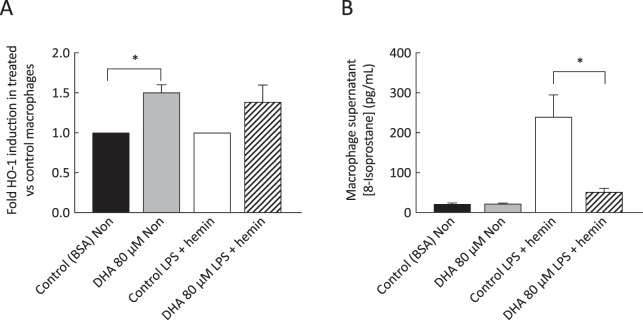
Increase in HO-1 transcripts and decrease in 8-isoprostane concentration in monocyte-derived macrophages from control participants. Real-time quantitative PCR, with raw data normalised to GAPDH, indicated increased HO-1 expression levels in DHA-supplemented non-stimulated macrophages and a trend for increase in LPS-stimulated macrophages exposed to DHA (**A**). In parallel, significant decreases were observed in 8-isoprostane concentrations in supernatants from hemin- and LPS-stimulated macrophages (**B**). Values are mean ± SEM of duplicate determinations (n = 5). *p < 0.05.

DHA supplementation induced a 50% increase in HO-1 mRNA in non-stimulated macrophages (p < 0.001) and a trend for increase (39%) was observed in macrophages exposed to hemin and LPS (Fig. 4A). The induction of HO-1 in macrophages stimulated with hemin and LPS was accompanied by decreases in concentrations of 8-isoprostane in supernatants from these cells (p < 0.01; Fig. 4B).

### DHA, SnPP and AH7614 did not adversely affect cell viability

U937 cell viability was determined in LPS-stimulated cells pre-treated with DHA and/or SnPP or AH7614 using the MTT assay. No adverse change in cell viability was detected for any of the treatment conditions at the concentrations used in this study (Table 1).

**Table 1:** Effect of DHA, SnPP and AH7614 on cell viability.

Compound	LPS-stimulated (% of control)
Albumin (control cells)	100
DHA 20 μM	109.04 ± 8.75
DHA 80 μM	99.71 ± 3.26
SnPP 10 μM	110.43 ± 4.80
SnPP 10 μM + DHA 20 μM	112.34 ± 6.63
SnPP 10 μM + DHA 80 μM	103.37 ± 5.91
AH7614 2 μM	110.58 ± 9.05
AH7614 2 μM + DHA 20 μM	120.52 ± 7.31
AH7614 2 μM + DHA 80 μM	105.12 ± 7.82

Abbreviations: DHA – docosahexaenoic acid,

SnPP – tin protoporphyrin.

### The protective effect of DHA was partially reversed by the heme oxygenase inhibitor SnPP

**Figure 5:**
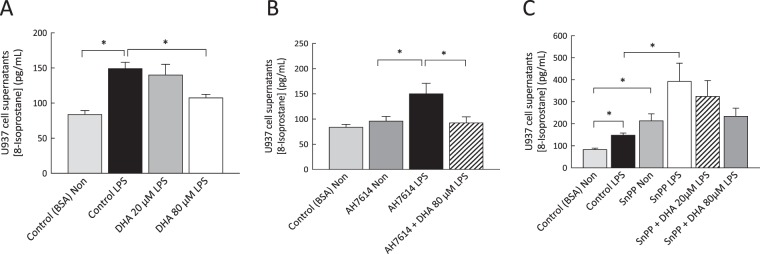
8-Isoprostane levels in non-stimulated and LPS-stimulated U937 cells incubated in the presence and/or absence of DHA (20 μM; 80 μM), the FFA4 antagonist AH7614 and the HO-1 inhibitor SnPP. Supplementation of cells with DHA 80 μM decreased 8-isoprostane production (**A**), a finding that was not reversed with AH7614 treatment (**B**). In LPS-stimulated cells treated with SnPP (**C**), no significant effect was observed for DHA at either of the concentrations tested. Data are expressed as mean ± SEM and are representative of at least six independent experiments. *p < 0.05.

DHA supplementation decreased 8-isoprostane production in LPS-stimulated U937 cells (Fig. 5A), an effect that was not reversed by pre-treatment of the cells with the FFA4 antagonist AH7614 (Fig. 5B). Pre-treatment of cells with the heme oxygenase inhibitor SnPP increased 8-isoprostane production to a level above that of LPS alone (Fig. 5C). A trend for reduced 8-isoprostane levels was observed in LPS-stimulated cells treated with SnPP and DHA.

### DHA exhibited modest free radical-scavenging activity

**Figure 6:**
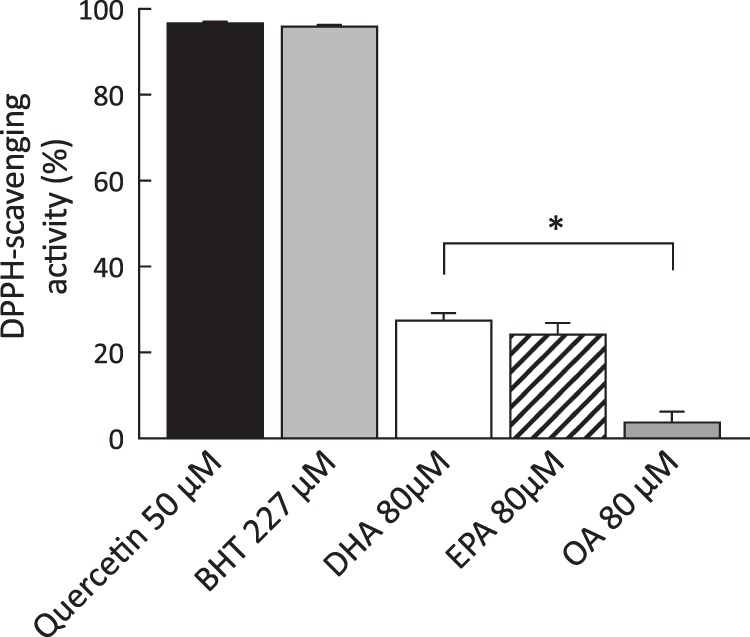
Free radical scavenging activity of quercetin 50 μM, BHT 227 μM, DHA 80 μM, EPA 80 μM and OA 80 μM as measured by colorimetric 1,1-diphenyl-2-picrylhydrazyl (DPPH) assay. Quercetin and BHT were included as positive controls. Absorbance values for the diluent (BSA) were subtracted from all data sets. Data are expressed as mean ± SEM and are representative of four independent experiments.

DHA and EPA exhibited modest scavenging activity for the stable DPPH free radical at 30 min (28% and 24% respectively) in comparison to positive controls (quercetin and BHT; Fig. 6). OA showed negligible free radical scavenging activity.

## Discussion

Macrophages feature prominently in human and experimental AAA formation and growth. The use of macrophages in this investigation contributed to the pre-clinical evaluation of n-3 PUFAs as a novel therapeutic strategy in AAA and uncovered mechanisms underlying n-3 PUFA-mediated improvements in oxidative stress status. Supplementation with the n-3 PUFA, DHA, suppressed production of 8-isoprostane, the gold standard biomarker of oxidative stress and a specific marker of lipid peroxidation^21^, in monocyte-derived macrophages obtained from patients with AAA. The results concord with data from both experimental animal studies^22,23^ and human clinical trials^24,25^ that suggest n-3 PUFAs decrease 8-isoprostane levels and ease oxidative stress. Importantly, the decrease observed in levels of 8-isoprostane in supplemented monocyte-derived macrophages suggests highly unsaturated n-3 PUFAs such as DHA do not increase susceptibility to lipid peroxidation. This contention is supported by the results of a pre-clinical study that indicated n-3 PUFA-mediated improvements in murine redox status were a consequence of reduced omega-6 fatty acid peroxidation and diversion away from the peroxyl radical pathway of F_2_-isoprostane formation^26^. It is noteworthy that the LPS-stimulated 8-isoprostane response in AAA macrophages was suppressed compared to control macrophages. This finding is in line with our previous report of an abrogated cytokine response to LPS in a AAA patient cohort, consistent with endotoxin tolerance^27^.

8-isoprostanes are stable, robust prostaglandin-like molecules formed independently of cyclooxygenase enzymes as a consequence of reactive oxygen species (ROS)-mediated peroxidation of esterified arachidonic acid^28^. Isoprostanes are released to their free acid form by phospholipase action and the activity of platelet-activating factor acetylhydrolase following their *in situ* generation from arachidonic acid^29^. Available evidence suggests quantification of these compounds accurately assesses total F_2_-isoprostane production and provides a highly precise time-integrated index of oxidation status^21^.

In addition to favourable impacts on multiple aspects of the oxidative stress response, n-3 PUFAs are well documented to down-regulate pro-inflammatory processes^30,31^ and to quench injury-, infection- and exercise-induced inflammatory conditions^32,33^. The mechanism for the anti-inflammatory effects of n-3 PUFAs has been attributed to activation of FFA4 receptors, resulting in preservation of inhibitor of κB (IκB) and prevention of nuclear translocation of NFκB^11^. Our data showed DHA-supplemented macrophages from both control participants and patients with AAA produced lower levels of the pro-inflammatory cytokines TNF-α and IL-6 in response to a pro-inflammatory stimulus. The anti-inflammatory and resolution-directed activities described for DHA represent an indirect mechanism by which n-3 PUFAs lower endogenous ROS and positively impact cellular redox status. Activated leukocytes are known to generate powerful oxidants during phagocytosis and to propagate a pro-oxidant milieu by producing cytokines (TNF-α, IL-6) that stimulate ROS generation by endothelial cells and other leukocytes^34^. Omega-3 PUFA interference with pro-inflammatory pathways reduces leukocyte activity and decreases leukocyte-mediated ROS production to favourably impact oxidative stress status^35^.

This study examined more direct mechanisms underlying n-3 PUFA-mediated improvements in oxidative stress by evaluating their impact on antioxidant enzymes and determining their capacity to scavenge ROS. Evidence of increased activity levels of GPx was found in non-stimulated macrophages treated with DHA in addition to increased levels of HO-1 mRNA in non-stimulated cells (50% increase) and a trend for increased levels in LPS-stimulated cells (39% increase). To investigate mechanisms underlying DHA-mediated suppression of 8-isoprostane production, additional experiments were carried out in a macrophage cell line. In these experiments, exposure of U937 cells to the FFA4 receptor antagonist AH7614 failed to reverse the suppressive effects of DHA on macrophage 8-isoprostane formation. This finding suggests that the FFA4 receptor is not the effector protein in DHA-mediated improvements in oxidative stress status. In further experiments, exposure of U937 cells to the heme oxygenase inhibitor SnPP increased 8-isoprostane production to a level above that of LPS alone and a trend for reduced 8-isoprostane levels was observed in LPS-stimulated cells treated with SnPP and DHA. Failure of the HO-1 inhibitor to fully reverse the protective effects of DHA suggests that n-3 PUFAs decrease oxidative stress through multiple pathways. In support of this, and in synergism with their positive impacts on inflammation and cytoprotective enzymatic antioxidants, n-3 PUFAs were shown to exhibit a modest capacity to scavenge free radicals (DHA, 28%; EPA, 24%) in comparison to oleic acid, which had negligible free radical scavenging activity, and the positive controls quercetin and BHT which had high levels of free radical scavenging activity.

While an *in vitro* cell culture system is subject to inherent limitations, our use of native, non-immortalised and non-transformed primary cells allowed a biologically relevant representation of the unique responses of AAA and control macrophages to supplementation with DHA. It is of note that the results obtained for 8-isoprostane and GPx in supplemented monocyte-derived AAA macrophages mirror the results of a recent n-3 PUFA supplementation trial conducted by our group in a cohort of AAA patients (unpublished findings). While clinical evidence regarding n-3 PUFA efficacy in AAA patient populations is limited, low serum concentrations of n-3 PUFAs have been associated with larger aneurysm size and a faster growth rate in a Japanese AAA cohort^36^. In addition, animal studies using an angiotensin II-infused ApoE^−/−^ mouse model have reported decreased oxidative stress signalling and reduced matrix metalloproteinase expression following n-3 PUFA supplementation^37,38^. Further long-term clinical studies will be required to determine whether n-3 PUFA-mediated improvements in oxidative stress translate into positive alterations in the histopathologic appearance of AAA at the level of the aorta.

Taken together, the findings of this study suggest the antioxidant potential of n-3 PUFAs arises from their ability, via multiple means, to lower endogenous ROS levels and positively impact cellular redox status. In addition to direct ROS scavenging, n-3 PUFAs suppress pro-inflammatory cytokine signalling and induce and/or activate cytoprotective antioxidant enzymes. Given the critical role of macrophages in the development and progression of AAA disease, the positive impact of DHA on macrophage pro-inflammatory responses serves as a stimulus for further investigation of this fatty acid in patients with AAA.

## Materials and Methods

### Participants

Patients with AAA were recruited from Sunshine Coast public and private clinics (Table 2). Control participants included members of the University of the Third Age and the general population of the Sunshine Coast, Queensland. The study was approved by the University of the Sunshine Coast (A/13/473 and A/16/833) and the Prince Charles Hospital Human Research Ethics Committees (HREC/12/QPCH/13). All experiments were performed in accordance with relevant guidelines and regulations. Written informed consent was obtained for each participant and all consented participants completed the study. The patient group included 19 men with AAA (mean maximal diameter 37.8 ± 5.3 mm) and the control group included 41 men without a documented AAA. AAA is known to be much more common in men than in women^39^ and male gender is associated with a 4-fold higher risk of AAA^40^. In addition, we have reported recently that differences exist in erythrocyte fatty acid incorporation for men and women^41^. While cells were obtained from all participants, limited supernatant volumes precluded their inclusion in all experimental assays. Aneurysm size, evaluated as part of routine care and AAA surveillance, was confirmed with transabdominal ultrasound. Exclusion criteria for AAA patients and control participants included: age below 60 or above 86 years, BMI above 39 kg m^−2^, uncontrolled hypertension, cardiac arrhythmia, heart failure, symptomatic aortic stenosis, chronic obstructive pulmonary disease, chronic inflammatory disease and regular use of prescription anti-inflammatory medication. A family history of AAA or known aneurysmal disease served as additional exclusion criteria for control participants. Dietary and medical history questionnaires were used to ensure adherence to inclusion/exclusion criteria. All participants refrained from non-prescribed anti-inflammatory medications 72 h prior to blood collection and abstained from alcohol and caffeine for the 12 h leading up to their study visit.

**Table 2:** Demographic, biometric and medical characteristics of AAA patients and control participants (mean ± SD).

Variable	AAA Patients (n = 19)	Control Participants (n = 41)
Age (years)	74.6 ± 5.8	71.9 ± 5.1
Smoking:		
Never	7 (37%)	17 (41%)
Past	10 (53%)	24 (59%)
Current	2 (11%)	0 (0%)
BMI (kg.m^2^)	26.9 ± 2.9	26.0 ± 4.1
Systolic blood pressure (mmHg)	136.5 ± 13.6	137.92 ± 13.2
Diastolic blood pressure (mmHg)	78.6 ± 6.1	79.4 ± 8.3
Hypertension	12 (63%)	15 (37%)
Diabetes	2 (11%)	0 (0%)
Coronary heart disease^†^	12 (63%)	4 (10%)
Medication		
Low-dose aspirin	5 (26%)	3 (7%)
Anti-hypertensive agents:		
Beta blockers^†^	8 (42%)	5 (12%)
Angiotensin II receptor antagonists	6 (32%)	7 (17%)
ACE inhibitors	2 (11%)	3 (7%)
Calcium channel blockers	2 (11%)	3 (7%)
Diuretics	2 (11%)	2 (5%)
Statins^†^	14 (74%)	13 (32%)

^†^Significantly different to control (Fisher’s exact test, p < 0.05).

AAA, abdominal aortic aneurysm; BMI, body mass index; NSAIDs, non-steroidal anti-inflammatory drugs.

### Fatty acid preparation

Fatty acids used in experiments included docosahexaenoic acid (C22:6n-3; DHA), eicosapentaenoic acid (C20:5n-3; EPA) and oleic acid (C18:1n-9; OA). Stock solutions (15 mM) of the fatty acids were prepared as previously described^42^. Control cells were supplemented with bovine serum albumin (BSA).

### U937 cell culture and cell viability assay

Human myelo-monocytic lymphoma U937 cells (American Type Culture Collection, USA) were used in this study to uncover mechanisms underlying n-3 PUFA-mediated alterations in inflammatory and oxidative stress status. Immortalised macrophage-like cell lines offer the advantages of ready availability and extensive proliferative potential and use of these cells allowed cellular responses to treatments to be studied under controlled conditions. U937 cells were maintained in RPMI 1640 medium (Sigma, USA) supplemented with 10% foetal calf serum (FCS; Gibco, USA), 1000 U/ml penicillin (Gibco, USA), and 1000 μg/ml streptomycin (Gibco, USA) in 25 cm^2^ or 75 cm^2^ tissue culture flasks (Corning, USA) at 37 °C humidified atmosphere with 5% CO_2_ and sub-cultured every 4^th^ or 5^th^ day. Following growth to confluence, cells were seeded onto 24-well culture plates (Corning Costar, USA) at a density of 4 × 10^5^ cells/well and differentiation was induced by exposure of cells to phorbol 12-myristate 13-acetate (PMA; Sigma, USA; 20 nM; 24 h). Cells were allowed to mature for a further 46–48 h in fresh complete medium prior to a 2 h exposure to the FFA4 receptor antagonist AH7614 (2 μM, n = 6) or the heme oxygenase inhibitor tin protoporphyrin (SnPP; 10 μM, n = 9). These concentrations of agents would be anticipated to inhibit 96.2% of FFA4 receptors^43^ and 99.9% of HO-1^44^. Cells were incubated with media containing bovine serum albumin (80 μM, BSA, control cells), EPA (20 μM, n = 6; 80 μM, n = 6) or DHA (20 μM, n = 6; 80 μM, n = 6) for a further 2 h. Treated U937 cells were exposed to lipopolysaccharide from *Escherichia coli* (LPS; 0.1 μg/ml, Serotype 0111:B4; Sigma, USA) for 6 h followed by collection and centrifugation (10,000 × g, 5 min, 4 °C) of the supernatants. Supernatants used to measure 8-isoprostane were preserved with butylhydroxytoluene (BHT; 2.5 mg/ml) to prevent artefactual 8-isoprostane formation. Cell viability was measured after treatment with AH7614 and SnPP using a commercially available colorimetric Cell Proliferation (MTT) Kit (Roche Diagnostics, Germany) in accordance with manufacturer’s instructions.

### Monocyte isolation protocol

Whole blood was collected in ethylenediaminetetraacetic acid (EDTA) tubes (4 × 6 ml) and centrifuged (400 × g, 25 min, 18 °C) with the brake turned off. The buffy coat was collected and diluted with 8 ml Ca^2+^/Mg^2+^-free Dulbecco’s phosphate buffered solution containing 1 mM EDTA (DPBS, pH 7.1) and centrifuged (150 × g, 18 °C, 10 min, brake off). The wash was repeated twice and the final spin was followed by removal of the supernatant and resuspension of cells with 1 ml of DPBS. Resuspended cells were layered to the surface of 2 ml aliquots of Ficoll-Paque Premium 1.073 (GE Healthcare, Sweden), the preparation was centrifuged (400 × g, 30 min, 18 °C) and cells within a dense white mononuclear band were collected and diluted with 5 ml DPBS. The cells were pelleted by centrifugation (500 × g, 10 min, 18 °C) and the supernatant was discarded. Monocyte purification was achieved by centrifugation with hyperosmotic Percoll Plus (Sigma, USA; prepared as per^45^) or by discontinuous density centrifugation using isotonic Percoll Plus at densities 1.070 g/ml, 1.062 g/ml, 1.060 g/ml and 1.058 g/ml. The Percoll preparation was centrifuged (400 × g, 15 min, 21 °C) and monocytes within a band located at the interface of the Percoll solution and the DPBS/media were collected and diluted with 4 ml DPBS. The cells were pelleted by centrifugation (550 × g, 10 min, 21 °C) and the supernatant was discarded. An aliquot of the cell pellet (1 μl) was stained with May-Grunwald stain (Merck Millipore) to confirm the presence of monocytes. Monocytes were resuspended in 1 ml serum-free complete Iscove’s Modified Dulbecco’s Medium (IMDM) supplemented with 1000 U/ml penicillin (Gibco, USA), 1000 μg/ml streptomycin (Gibco, USA) and 2 mM L-glutamine (Gibco, USA) and cell number was determined using a haemocytometer.

### Autologous serum preparation

Whole blood from each participant was collected in serum separator tubes (2 × 6 ml) and allowed to clot at 22 °C for 30 min prior to centrifugation (1500 × g, 15 min, 15 °C). The serum was collected, centrifuged (4000 × g, 5 min, 4 °C) and the supernatant was stored at −80 °C for use as an autologous serum supplement in cell cultures of isolated monocytes.

### Monocyte culture

Monocytes were seeded onto 24 well culture plates (Corning Costar, USA) at a density of 3 × 10^5^ cells/well (final volume 600 μl/well). Plated cells were transferred to a 37 °C humidified 5% CO_2_ incubator for a period of 2 h, at which time the supernatant containing non-adherent cells was removed following gentle flushing and the medium was replaced with IMDM containing 5% autologous serum (v/v) and 50 ng/ml macrophage colony-stimulating factor (M-CSF, Sigma, USA). Further media changes occurred on days 1 and 4.

### Macrophage maturation and activation

Monocytes matured into a morphologically heterogeneous macrophage population over 7 days. On day 7, the medium (IMDM and all additives except M-CSF) was replaced for a final time and the adherent macrophages were exposed to a 1 h treatment with BSA (control cells), DHA (20 μM or 80 μM) or OA (80 μM). The fatty acid concentrations used in the supplementation of cells have been documented to have no adverse effects on cell viability^46,47^ and have been shown to approximate the plasma DHA concentration, previously reported to be in the range of 91–122 μM^48^. To measure 8-isoprostane, treated cells were polarised toward an M1 phenotype through exposure to 20 ng/ml interferon gamma (IFN-*γ*) and 0.1 μg/ml LPS for a further 24 h. LPS is a well-characterised activator of TLR4, which is known to be expressed in human macrophages. Elevated LPS levels have been associated with some AAA cases^49,50^, although this association remains a contentious issue^51,52^. All supernatants were collected, centrifuged (10,000 × g, 5 min, 4 °C) and aliquoted with and without BHT (2.5 mg/ml). For glutathione peroxidase (GPx) activity assays, a 300 μl aliquot of GPx collection buffer (50 mM Tris-HCl, pH 7.5, 5 mM EDTA, 1 mM DTT) was added to each well and adherent cells were harvested and stored at −80 °C. For catalase activity assays, a 300 μl aliquot of catalase collection buffer (50 mM KH_2_PO_4_, pH 7.0, containing 1 mM EDTA) was added to each well and adherent cells were harvested and stored at −80 °C.

### Measurement of free 8-isoprostane

To establish oxidative stress status, free 8-isoprostane levels were measured in BHT-preserved U937 and monocyte-derived macrophage supernatants without further manipulation using a commercially available enzyme immunoassay kit (Cayman Chemical Company, Ann Arbor, USA) in accordance with manufacturer’s instructions. Samples were stored at −80 °C and assayed within 30 days of collection.

### Cytokine assays

Cytokines (IL-6, TGF-β, TNF-α, IL-1β, IL-10) were measured in monocyte-derived macrophage supernatants using commercially available enzyme immunoassay kits (Affymetrix eBioscience, San Diego, USA) in accordance with manufacturer’s instructions.

### Antioxidant enzyme activity assays

GPx activity and catalase enzymatic activity was measured in monocyte-derived macrophage lysates using commercially available kits (Cayman Chemical Company, Ann Arbor, USA) in accordance with manufacturer’s instructions. Enzyme activity was expressed per μg protein^53^.

### Free radical-scavenging activity assay

The free radical-scavenging activities of 80 μM DHA, EPA and OA were examined using a colorimetric 1,1-diphenyl-2picrylhydrazyl (DPPH) assay as previously described^54^. Briefly, fatty acids and positive controls (BHT 227 μM; quercetin 50 μM) were incubated with methanolic DPPH solutions (100 μM; 30 min; 22 °C) and absorbance was measured at 518 nm. A methanolic solution of DPPH (100 μM), decayed in the presence of 0.5 mM ascorbic acid to allow clearance of colour, was used for background correction. Results were expressed as percentage of a negative control using the following equation:

$$\mathrm{DPPH}-\mathrm{scavenging}\,{\rm{activity}}\,( \% )={\rm{100}}-[(\frac{\mathrm{Blank}-\mathrm{adjusted}\,{\rm{absorbance}}\,{\rm{of}}\,{\rm{sample}}}{\mathrm{Blank}-\mathrm{adjusted}\,{\rm{absorbance}}\,{\rm{of}}\,{\rm{negaticve}}\,{\rm{control}}})\times {\rm{100}}]$$

### RNA isolation and real-time quantitative PCR

Human blood derived monocytes were harvested and cultured as before. On day 7 of culture, adherent macrophages were exposed to a 1 h treatment with albumin (80 μM, control cells) or DHA (80 μM). Treated cells were incubated with hemin (20 μM; Sigma) and stimulated with IFN-*γ* (20 ng/ml) and LPS (1 μg/ml) for 24 h to provoke super production of HO-1^55^. Supernatants were collected, centrifuged and aliquoted as before. Cells were rinsed with DPBS and a 300 μl aliquot of RNA*Later* RNA Stabilisation Reagent (Qiagen) was added to each well. Adherent cells were harvested by scraping and cells were cooled at 4 °C for 24 h followed by transfer to −20 °C for storage until analysis. Total RNA was extracted from samples using the Isolate II RNA mini kit (Bioline) prior to generation of cDNA using the SensiFAST cDNA synthesis kit (Bioline) and amplification of samples against transcripts by real-time quantitative PCR using the SensiMix SYBR No-Rox kit (Bioline) and a Rotor-Gene Q thermal cycler (Qiagen). Primers were from Sigma and included HO-1 forward 5′CCAGCAACAAAGTGCAAGATTC 3′, HO-1 reverse 5′TCACATGGCATAAAGCCCTACAG 3′ ^56^ and GAPDH forward 5′GGGGGAGCCAAAAGGGTCATCATCT 3′, GAPDH reverse 5′GAGGGGCCATCCACAGTCTTC3′ ^57^. No template controls were run in each reaction and no contamination was observed. Normalisation points included a) spectrophotometric RNA quantification (Nanodrop, ND100), b) inclusion of a reference sample in each run and c) determination of mRNA levels of an internal control (GAPDH) for all samples. Quantitation of relative gene expression was based on the comparative threshold cycle method (2^−ΔΔCt^)^58^ with raw data normalised to GAPDH.

### Data analysis

Continuous demographic variables were compared using a student’s t-test and are presented as mean ± SD. Categorical demographic variables were compared using a Fisher’s exact test and data are presented as number (percentage). Experimental data are presented as mean ± SEM. Data were assessed to determine normality (Shapiro-Wilk test) and homogeneity of variance (Levene statistic). Where data followed Gaussian distribution, data were assessed using a student’s t-test, assuming equal or unequal variance as determined by the Levene statistic. Where the null hypothesis for the Shapiro-Wilk test was rejected, data were analysed using the Mann Whitney U test. Outliers in macrophage supernatant cytokine data (TNF-α n = 2, IL-6 n = 3, IL-10 n = 2 and IL-1 β n = 2) were identified using the extreme studentised deviate many-outlier procedure^59^ and excluded from analysis. For U937 experiments, significance was determined using one-way ANOVA corrected for multiple comparisons with Sidak test. Statistical significance was set at p < 0.05.
